# [60]Fullerene derivative modulates adenosine and metabotropic glutamate receptors gene expression: a possible protective effect against hypoxia

**DOI:** 10.1186/s12951-014-0027-7

**Published:** 2014-08-14

**Authors:** Davide Giust, Tatiana Da Ros, Mairena Martín, José Luis Albasanz

**Affiliations:** 1 Departamento de Química Inorgánica, Orgánica y Bioquímica, Facultad de Ciencias y Tecnologías Químicas, Centro Regional de Investigaciones Biomédicas, Universidad de Castilla-La Mancha, Ciudad Real, Spain; 2 Institute of Inflammation and Repair, University of Manchester, Manchester, UK; 3 Dipartimento di Scienze Chimiche e Farmaceutiche, Università degli Studi di Trieste, Trieste, Italy; 4 Departamento de Química Inorgánica, Orgánica y Bioquímica. Facultad de Medicina de Ciudad Real, Centro Regional de Investigaciones Biomédicas, Universidad de Castilla-La Mancha, Ciudad Real, Spain

**Keywords:** Fullerenes, Adenosine receptors, Metabotropic glutamate receptors, Hypoxia, Neuroprotection

## Abstract

**Background:**

Glutamate, the main excitatory neurotransmitter, is involved in learning and memory processes but at higher concentration results excitotoxic causing degeneration and neuronal death. Adenosine is a nucleoside that exhibit neuroprotective effects by modulating of glutamate release. Hypoxic and related oxidative conditions, in which adenosine and metabotropic glutamate receptors are involved, have been demonstrated to contribute to neurodegenerative processes occurring in certain human pathologies.

**Results:**

Human neuroblastoma cells (SH-SY5Y) were used to evaluate the long time (24, 48 and 72 hours) effects of a [60]fullerene hydrosoluble derivative (t3ss) as potential inhibitor of hypoxic insult. Low oxygen concentration (5% O_2_) caused cell death, which was avoided by t3ss exposure in a concentration dependent manner. In addition, gene expression analysis by real time PCR of adenosine A_1_, A_2A_ and A_2B_ and metabotropic glutamate 1 and 5 receptors revealed that t3ss significantly increased A_1_ and mGlu_1_ expression in hypoxic conditions. Moreover, t3ss prevented the hypoxia-induced increase in A_2A_ mRNA expression.

**Conclusions:**

As t3ss causes overexpression of adenosine A_1_ and metabotropic glutamate receptors which have been shown to be neuroprotective, our results point to a radical scavenger protective effect of t3ss through the enhancement of these neuroprotective receptors expression. Therefore, the utility of these nanoparticles as therapeutic target to avoid degeneration and cell death of neurodegenerative diseases is suggested.

## Introduction

Low oxygen availability in neuronal cells is the main cause of cognitive and physical deficiencies in patients who suffered ischemic toxicity in brain. Even though various factors are involved during the partial decrease of oxygen concentration in cells, the activation of specific factors named HIFs (hypoxia inducible-factors), which induce the transcription of specific genes involved in several transduction pathways, mainly happen at low concentrations of oxygen in cells [1]. The consequent oxidative stress has been related to cell death processes, generally apoptotic, upon the formation of reactive oxygen species (ROS) and subsequently oxidative modifications of lipid, DNA, cell membrane proteins and other target molecules. The oxidative stress has been furthermore related to the development of some neurodegenerative diseases as Alzheimer’s and Parkinson’s [2]. The oxidative damage by free radicals is known to be originated in mitochondrial respiratory chain by complex I and III as source of superoxide anion (O_2_^−^) [3]. However, these organelles have protective antioxidant enzymes that convert reactive species into no toxic ones [4]. Some of these enzymes belong to the family of superoxide dismutase (SOD), converting superoxide anion into H_2_O_2_; catalase, converting H_2_O_2_ into water, and other enzymes like glutathione reductase, whose expression is induced by ROS. Nevertheless some enzymes may change their function from antioxidant to oxidant, mainly due to the increase of iron and copper linked to brain aging [2]. Thus a dramatic increase in ROS has been related to cell death induced by oxidative stress and to neurodegenerative diseases as Alzheimer’s [5]. Apoptotic cell death is mainly mediated by an increase in mitochondrial permeability to calcium which promotes cytochrome c release [6]. In that sense, it has been proposed that the first step of Alzheimer’s disease is the oxidative damage originated by ROS [7].

Adenosine and metabotropic glutamate receptors (mGluR) have been largely studied for their implication in hypoxic injury. It has been repeatedly shown that adenosine can protect tissues against the negative consequences of hypoxia or ischemia [8],[9], mainly by acting on the A_1_AR. Hence, survival after a hypoxic challenge may be reduced if A_1_ARs are absent or blocked [10]. Because the A_2B_AR promoter contains a functional binding site for hypoxia-inducible factor, the onset of hypoxia strongly induces its expression [11]. Moreover, a tissue protective role of A_2B_AR signaling during hypoxic conditions has been reported [12]. On the other hand, it has been described the role of mGluRs during hypoxic conditions. The protective effect of mGluRs of group I and the involvement of protein kinase C during hypoxic conditions have been reported [13]. In addition, Opitz and co-workers described in rat hippocampal slices dissimilar responses to hypoxic conditions depending on different group of mGluRs involved. Thus, the activation of group II was found to decrease cell recovery from hypoxia. However, a beneficial role of pre-activation of group I, as well as their detrimental role if activated during hypoxic conditions, was reported [14].

Fullerene derivatives are effective redox-active compounds towards ROS such as superoxide anion radical (O_2_^−^), hydroperoxide (ROOH), and hydroxyl radical (^•^OH) produced during oxidative stress and responsible for different type of cell damages [15]. These properties are peculiar of fullerenes because of their general low energy of LUMO (Lowest Unoccupied Molecular Orbital) towards a high HOMO (Highest Occupied Molecular Orbital) level, conferring them radical quenching properties. For example, a polyhydroxylated C_60_ (fullerenol) was reported to have quenching activity for O_2_^−^[16]. Furthermore, it has been demonstrated that fullerenol presents a decreased ROS quenching activity with respect to the parent C_60_ since conjugated double bonds are widely broken. Thus, experiments with C_60_-dimalonic acid, having properties rather similar to C_60_, demonstrated its major efficacy in quenching radical species. This activity seems to be related to anti-aging and neuroprotective effect of fullerene derivatives [17],[18]. Therefore, [60]fullerene may be consider as the ideal candidate for protective properties in living systems subjected to oxidative stress even though the functionalization of [60]fullerene, used to increase its solubility in aqueous systems for better interfacing with biologic systems, is often accomplished with a partial loss of its antioxidant activity. Nevertheless, it was demonstrated that the bis-functionalization of [60]fullerene by means of azomethine ylide 1,3 dipolar cycloaddition leading to bis-pyrrolidine derivatives, confers a certain electronic stability to the fullerene carbon cage without depriving its quenching properties towards radical species [19]. In that sense, a series of highly hydrosoluble [60]fullerene bis-adducts were synthesized and encouraging results on assays in biological substrates have been obtained [20]. The aim of the present work was to study the possible protective effect of the *trans*-3 isomer of [60]fullerene (t3ss) in SH-SY5Y cells subjected to hypoxic conditions and the involvement of adenosine and metabotropic glutamate receptors in this t3ss-mediated effect.

## Materials and methods

### Materials

**Figure 1:**
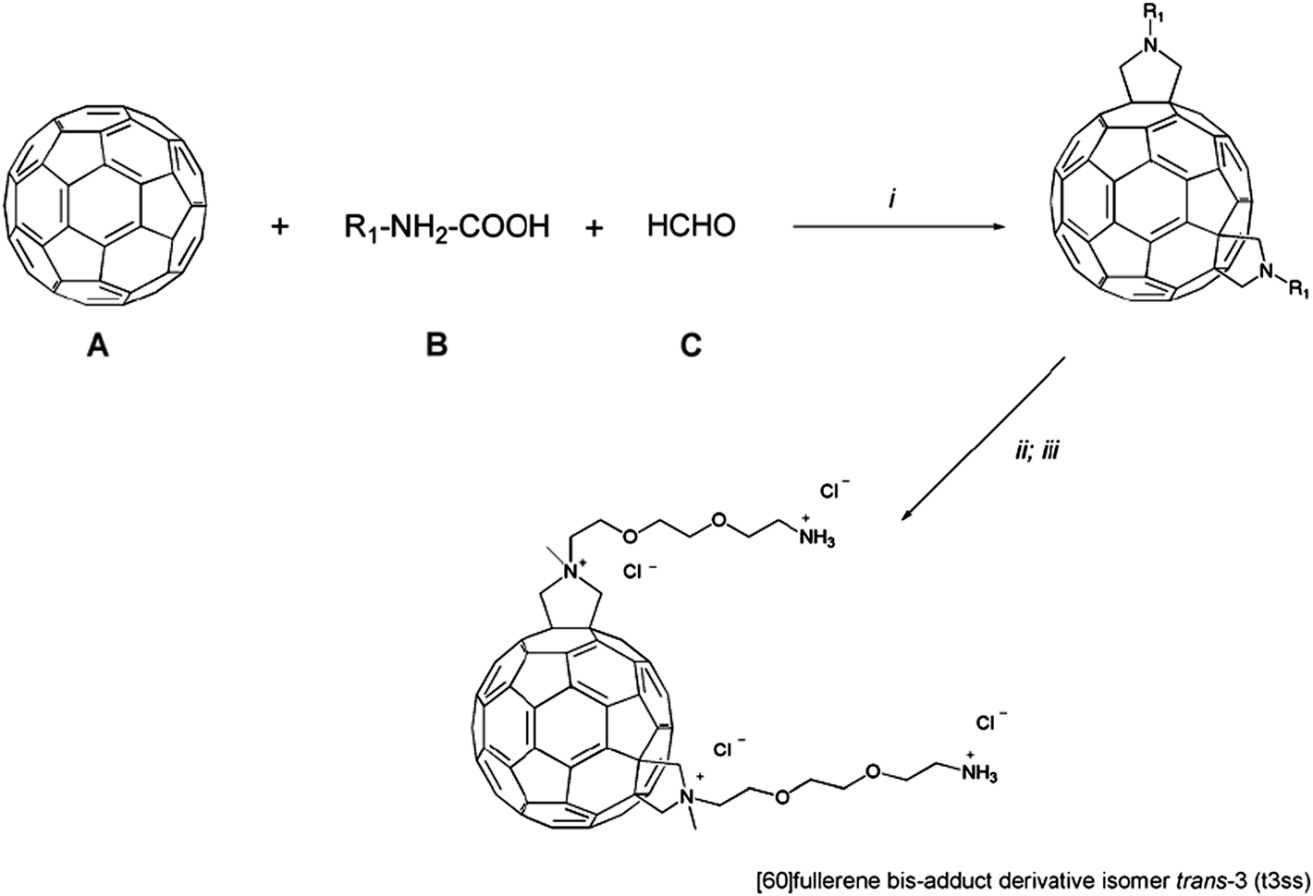
**Synthetic scheme for** ***trans-3*** **isomer [60]fullerene bis-adduct derivative (t3ss).** R_1_ = CH_2_CH_2_OCH_2_CH_2_OCH_2_CH_2_NHBoc. Reagent and conditions: (*i*) molar ratio A:B:C 1:2:5, toluene, 2 h at reflux, isolation of trans-3 isomer among the reaction mixture; (*ii*) CH_3_I, CH_2_Cl_2_, 24 h at 80°C; (*iii*) HCl (g), CH_3_OH, 20 min at 0°C. For more details and procedures see [20].

The hydrosoluble [60]fullerene bis-adduct *trans*-3 (t3ss) was synthesized as described in Figure 1. All reagents and solvents employed were from Sigma Aldrich (Germany). For an exhaustive reference of the product synthesis see [20].

### Cell culture

Human neuroblastoma SH-SY5Y cells (acquired from American Type Cell Collection) were grown in DMEM (Dulbecco’s Modified Eagle’s Medium) supplemented with 10% fetal bovine serum, and 1% of mixture antibiotic-antimycotic (Gibco, USA), humidified atmosphere with 5% CO_2_ at 37°C. SH-SY5Y cells were subcultured (passages 3–12) on 10 ml Petri dish (Nunc, Denmark). At confluence, they were detached by Trypsin (Tryple Express, Gibco, USA), centrifuged and the cells re-suspended in complete growth medium and plated onto 24- or 96-well dishes (Nunc, Denmark) to have a final density per well of 2 × 10^5^ and 3 × 10^4^ cells, respectively.

### MTT reduction assay

Cell viability was determined using an in vitro toxicology assay kit based on the reduction of 3-[4,5-dimetylthiazol-2-yl]-2,5-diphenyltetrazolium bromide (MTT) purchased from Sigma (Madrid, Spain), according to Mosmann [21]. Briefly, SH-SY5Y cells were seeded at 3 × 10^4^ cells per well in 96-well dishes and exposed to hypoxic conditions (95% N_2;_ 5% O_2_) for desired period (6, 24, 48 and 72 h) and treated with different t3ss concentrations (25–150 μM). At the end of hypoxic insults or fullerene derivative treatment, cells were incubated in culture medium with MTT solution (5 mg/mL) at 37°C for 3 h. After incubation, MTT solubilization solution (10% Triton X-100 plus 0.1 N HCl in anhydrous isopropanol) was added to the wells to dissolve formazan crystals. The plates were thoroughly shaken and the absorbance of each well was measured at 570 nm.

### Total RNA isolation and preparation of cDNA

Total RNA was extracted from cells using an ABI 6100 Nucleic Acid PrepStation and chemicals according to the manufacturer’s protocol (Applied Biosystems, Foster City, CA). Ratio of A_260_/A_280_ (RNA purity) was in the range 1.9-2.1. RNA concentrations were determined from the A_260_. Total RNA was isolated and stored at −80°C. One microgram of total RNA was reverse transcribed using Applied Biosystems’ High-Capacity cDNA Archive Kit according to manufacturer’s protocol.

### Quantitative real time RT-PCR analysis

To assess relative gene expression in SH-SY5Y neuroblastoma cells, quantitative real time RT-PCR analysis [22] was performed with an Applied Biosystems Prism 7500 Fast Sequence Detection System, using TaqMan universal PCR master mix according to the manufacturer’s specifications (Applied Biosystems, Foster City, CA). The TaqMan probes and primers for A_1_ (assay ID: Hs00181231), A_2A_ (assay ID: Hs00386497), A_2B_ (assay ID: Hs00169123), mGlu_1_ (assay ID: Hs00168250), mGlu_5_ (assay ID: Hs00168275), and β-actin (assay ID: Hs99999903) were assay-on-demand gene expression products (Applied Biosystems). TaqMan® Gene Expression Assays all have an efficiency of 1.0, which means a doubling of PCR product in every cycle is guaranteed. The TaqMan primer and probe sequences are packaged together in a 20× solution. The sequences are proprietary, so they are not available. The gene-specific probes were labeled using reporter dye FAM. A non-fluorescent quencher and the minor groove binder were linked at the 3’ end of probe as quenchers. The thermal cycler conditions were as follows: hold for 20 s at 95°C, followed by two step PCR for 40 cycles of 95°C for 3 s followed by 60°C for 30 s. Levels of RNA expression were determined using the 7500 Fast System SDS software version 1.3.1 (Applied Biosystems) according to the 2^−ΔΔCt^ method. Briefly, expression results of a gene were normalized to endogenous control β-actin relative to a calibrator (normoxia sample), consisting of the mean expression level of the receptor gene as follows: 2^−ΔΔCt^ = 2 ^− ((Ct receptor gene − Ct actin) *sample* − (Ct receptor gene − Ct actin) *calibrator*)^. β-actin is an appropriated endogenous control as its expression did not change after t3ss exposure [23],[24]. The results from 4–5 independent repeat assays, performed in different plates each using different cDNA’s from the cultures analyzed, were averaged to produce a single mean value for each mRNA.

### Statistical and data analysis

Data statistical analysis was performed using Student *t*-test and one-way ANOVA test and Dunnett post test with the GraphPad Prism 5 program (GraphPad Software, San Diego, CA, USA). Differences between mean values were considered statistically significant at p < 0.05.

## Results and discussion

**Figure 2:**
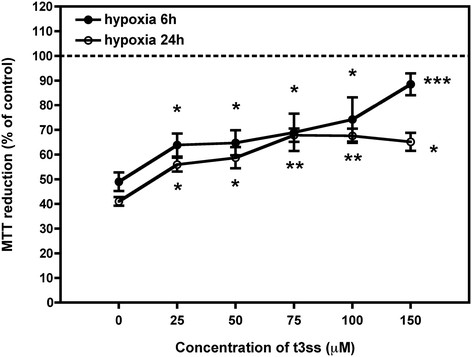
**MTT reduction viability assay on SH-SY5Y cells after hypoxic conditions (5% O**_**2,**_**95% N**_**2**_**) in the absence or the presence of t3ss derivative.** SH-SY5Y cells were subjected to 6 and 24 h of hypoxic condition in the presence or absence of different concentrations of [60]fullerene hydrosoluble t3ss derivative (t3ss) (25–150 μM). The obtained values are the average of three independent experiments, collected following MTT reduction assay protocol as described in *Methods*. The dotted line indicates the 100% of cells survival intended as cells growing in normal oxygen conditions (21% O_2_). *p < 0.05, **p < 0.01, ***p < 0.001 significantly different from cells survival in the absence of t3ss derivative according to Student *t*-test.

MTT assays showed a significant cell death after 6 and 24 h of hypoxic insult (5% O_2_). Interestingly, cell viability was strongly recovered when t3ss was present during hypoxia being this effect concentration dependent (Figure 2). This protective effect of t3ss was detected and very similar at both 6 and 24 hours hypoxic insult.

Next step was to test the possible role of t3ss on gene expression modulation of receptors involved in neurodegenerative processes and diseases, such as adenosine (AR) and metabotropic glutamate receptors (mGluR) [25]–[27]. Even though the treatment of SH-SY5Y cells with 75 μM t3ss derivative exhibited similar significant protective effect in both the 6 and 24 h period of hypoxia, as observed from MTT assay, in the shorter period of treatment, we did not observe any change in gene expression from qPCR (data not shown). Therefore, we assessed the effect of t3ss (75 μM) on gene expression for 24 h and longer periods (48 and 72 h) of exposure to hypoxic insult.

**Figure 3:**
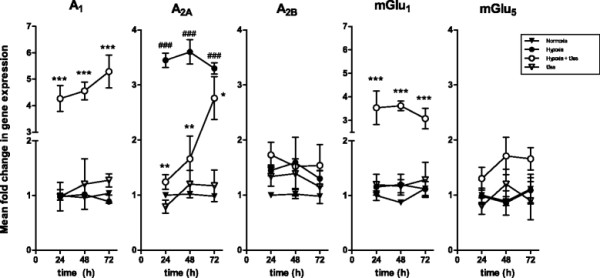
**Gene expression evaluated by quantitative real time-PCR in SH-SY5Y cells exposed to hypoxic and normoxic conditions.** SH-SY5Y cells were subjected normoxia and hypoxia (5% O_2_) during 24, 48 and 72 hours, in the absence and in the presence of 75 μM t3ss derivative. The corresponding RNA was analyzed by quantitative real time PCR, as described in *Methods*, using the following gene targets: adenosine A_1_, A_2A_, A_2B_ receptors, mGlu_1_ and mGlu_5_ receptors. Data are mean ± SEM of four to five independent experiments. ## p < 0.01 significantly different from the value at the corresponding time in normoxia; *p < 0.05, **p < 0.01 and ***p < 0.001, significantly different from the value at the corresponding time in hypoxia, according to ANOVA test and Dunnett post test.

Results from qPCR assays revealed a constant gene expression of all the receptors analyzed in the present study during the considered periods of time (24, 48 and 72 h) in normoxia (Figure 3). Only A_2A_ mRNA expression was significantly enhanced at 24 h and longer time in hypoxia, pointing out to a possible role of A_2A_ in the viability loss detected at 24 h. A slight not significant increase of A_2B_ expression was also detected, while the others genes analyzed were not modified by hypoxia at any time assayed.

Interestingly, although t3ss did not change the expression of the analyzed genes when applied in normoxia, it significantly reduced the hypoxia-induced increase in A_2A_ expression. Moreover, t3ss also increased A_1_ and mGlu_1_ expression after 24 h in hypoxia, being this effect maintained at 48 and 72 h. However, t3ss did not modify A_2B_ and mGlu_5_ gene expression during hypoxia.

These results show that the protective effect of [60]fullerene derivative against hypoxia, with increased effectiveness at higher concentrations, could be attributed, at least in part, to its modulatory effect on target receptor genes.

The neuroprotection induced by adenosine operates two well-known synaptic actions of A_1_ARs that also occur under normoxic conditions and are of particular relevance in the case of hypoxia: a decrease of neurotransmitter release via inhibition of presynaptic calcium entry through the blocking of calcium channels [28], and postsynaptically inhibiting calcium entry via inhibition of NMDA (N-methyl-D-aspartate) receptors [29]. In line with this, the observed increase in A_1_ARs gene expression by t3ss derivative, as it has been previously reported in SK-N-MC cells [23], could be related to the described protective role exerted by these receptors during hypoxic conditions [30].

Our group previously demonstrated the implication of A_1_ and A_2A_ receptors during hypoxic conditions in rat C6 glioma cells, where hypoxia (5% O_2_) caused a significant decrease in A_1_ receptors while A_2A_ receptors were significantly increased through a mechanism in which endogenous adenosine and tonic A_1_ receptor activation is involved [31]. On the other hand, it has been demonstrated that during oxidative stress and related toxicity, the use of antagonists of adenosine A_2A_ receptors may be considered a valid therapeutic approach [32]. Moreover, it has been reported that A_2A_ARs modulate glutamate uptake in cultured astrocytes and gliosomes [33] and stimulate glutamate release, thus promoting increased glutamate levels which could be excitotoxic mainly through NMDA receptors overactivation. However, protective effects related to survival of neurons have been attributed to the group I of metabotropic glutamate receptors, mainly mGlu_1_[34].

Fullerene derivatives may act on NMDA receptors and on the variation of Ca^2+^ homeostasis and inhibit the excitotoxic death, as it has been reported in cultured cortical neurons exposed to NMDA agonist [17]. The Ca^2+^ homeostasis has been reported to be also modulated by mGlu_1_ receptors as they regulate voltage dependent calcium channels [35]. Thus, the effect of t3ss derivative on the modulation of mGlu_1_ gene expression could be hypothetically related to the one reported for mGlu_1_ antagonists, which reduce the damage in post ischemic conditions [36]. The herein proposed possible modulatory effect of t3ss on NMDA receptors, and consequently on Ca^2+^ homeostasis could be related to the modulation of the expression of metabotropic glutamate receptor belonging to group I [37]. Both mechanisms of action of t3ss could thus lead to the protective effect observed during MTT viability assay, acting both synergically or independently one from the other.

In summary, results presented herein show for the first time that the modulation of adenosine and metabotropic glutamate receptors exhibited by a [60]fullerene derivative which could be, at least in part, responsible for its neuroprotective effect against hypoxic insults. Given that hypoxia and subsequent adenosine generation is likely an acute response to numerous injuries, including neurodegenerative diseases as Alzheimer’s, the modulation of adenosine and metabotropic glutamate receptors by t3ss during hypoxia could open new lines of research in the field of therapeutics.

## Conclusions

In the present paper SH-SY5Y cells were used as a model to hypoxic injury which caused cell death. Hypoxia has been related to neurodegeneration characteristic of many neurodegenerative diseases. Adenosine and metabotropic glutamate receptors have been shown to be impaired in Alzheimer’s disease and other neurodegenerative diseases. Results from this work show that a hydrosoluble fullerene derivative (t3ss) is able to avoid the cell death during hypoxia. In addition, fullerene derivative also increases adenosine A_1_ and metabotropic glutamate receptors expression during hypoxia. Therefore, our results suggest fullerenes as neuroprotective molecules and open new therapeutic perspectives to be considered to avoid degeneration and neuronal death which is related to neurodegenerative diseases. However, additional studies will be necessary in order to confirm this hypothesis.

## Competing interest

There is no conflict of interest including any financial, personal, or other relationships with other people or organizations.

## Authors’ contributions

DG carried out all experiments of viability and quantitative real time PCR. TR carried out the synthesis and purification of t3ss. MM and JLA conceived all the study, participated in its design and coordination and wrote the manuscript. All authors read and approved the final manuscript.

## References

[B1] Kenneth NS Rocha S Regulation of gene expression by hypoxia Biochem J 2008 414 19 29 10.1042/BJ20081055 18651837

[B2] Barnham KJ Masters CL Bush AI Neurodegenerative diseases and oxidative stress Nat Rev Drug Discov 2004 3 205 214 10.1038/nrd1330 15031734

[B3] Jezek P Hlavatá L Mitochondria in homeostasis of reactive oxygen species in cell, tissues, and organism Int J Biochem Cell Biol 2005 37 2478 2503 10.1016/j.biocel.2005.05.013 16103002

[B4] Yu BP Cellular defenses against damage from reactive oxygen species Physiol Rev 1994 7 139 162 8295932 10.1152/physrev.1994.74.1.139

[B5] De Leo ME Borrello S Passantino M Palazzotti B Mordente A Daniele A Filippini V Galeotti T Masullo C Oxidative stress and overexpression of manganese superoxide dismutase in patients with Alzheimer’s disease Neurosci Lett 1998 250 173 176 10.1016/S0304-3940(98)00469-8 9708860

[B6] Mattson MP Kroemer G Mitochondria in cell death: novel targets for neuroprotection and cardioprotection Trends Mol Med 2003 9 196 205 10.1016/S1471-4914(03)00046-7 12763524

[B7] Nunomura A Perry G Aliev G Hirai K Takeda A Balraj EK Jones PK Ghanbari H Wataya T Shimohama S Chiba S Atwood CS Petersen RB Smith MA Oxidative damage is the earliest event in Alzheimer disease J Neuropathol Exp Neurol 2001 60 759 767 11487050 10.1093/jnen/60.8.759

[B8] Gomes CV Kaster MP Tomé AR Agostinho PM Cunha RA Adenosine receptors and brain diseases: neuroprotection and neurodegeneration Biochim Biophys Acta 2011 1808 5 1380 1399 10.1016/j.bbamem.2010.12.001 21145878

[B9] Fredholm BB Adenosine and neuroprotection Int Rev Neurobiol 1997 40 259 280 10.1016/S0074-7742(08)60723-0 8989624

[B10] Johansson B1 Halldner L Dunwiddie TV Masino SA Poelchen W Giménez-Llort L Escorihuela RM Fernández-Teruel A Wiesenfeld-Hallin Z Xu XJ Hårdemark A Betsholtz C Herlenius E Fredholm BB Hyperalgesia, anxiety, and decreased hypoxic neuroprotection in mice lacking the adenosine A1 receptor Proc Natl Acad Sci U S A 2001 98 9407 9412 10.1073/pnas.161292398 11470917 PMC55434

[B11] Kong T Westerman KA Faigle M Eltzschig HK Colgan SP HIF-dependent induction of adenosine A_2B_ receptor in hypoxia FASEB J 2006 20 2242 2250 10.1096/fj.06-6419com 17077301

[B12] Koeppen M1 Eckle T Eltzschig HK Interplay of hypoxia and A2B adenosine receptors in tissue protection Adv Pharmacol 2011 61 145 186 10.1016/B978-0-12-385526-8.00006-0 21586359

[B13] Schröder UH Opitz T Jäger T Sabelhaus CF Breder J Reymann KG Protective effect of group I metabotropic glutamate receptor activation against hypoxic/hypoglycemic injury in rat hippocampal slices: timing and involvement of protein kinase C Neuropharmacology 1996 38 209 216 10.1016/S0028-3908(98)00180-4 10218861

[B14] Opitz T Richter P Carter AJ Kozikowski AP Shinozaki H Reymann KG Metabotropic glutamate receptor subtypes differentially influence neuronal recovery from in vitro hypoxia/hypoglycemia in rat hippocampal slices Neuroscience 1995 68 989 1001 10.1016/0306-4522(95)00195-O 8545005

[B15] Rajendran P Nandakumar N Rengarajan T Palaniswami R Gnanadhas EN Lakshminarasaiah U Gopas J Nishigaki I Antioxidants and human diseases Clin Chim Acta 2014 436C 332 347 10.1016/j.cca.2014.06.004 24933428

[B16] Mirkov SM Djordjevic AN Andric NL Andric SA Kostic TS Bogdanovic GM Vojinovic-Miloradov MB Kovacevic RZ Nitric oxide-scavenging activity of polyhydroxylated fullerenol, C60(OH)24 Nitric Oxide 2004 11 201 217 10.1016/j.niox.2004.08.003 15491853

[B17] Dugan LL Turetsky DM Du C Lobner D Wheeler M Almli CR Shen CK Luh TY Choi DW Lin TS Carboxyfullerenes as neuroprotective agents Proc Natl Acad Sci 1997 94 9434 9439 10.1073/pnas.94.17.9434 9256500 PMC23208

[B18] Bisaglia M Natalini B Pellicciari R Straface E Malorni W Monti D Franceschi C Schettini G C3-fullero-tris-methanodicarboxylic acid protects cerebellar granule cells from apoptosis J Neurochem 2000 74 1197 1204 10.1046/j.1471-4159.2000.741197.x 10693952

[B19] Guldi DM Prato M Excited-state properties of C(60) fullerene derivatives Acc Chem Res 2000 33 695 703 10.1021/ar990144m 11041834

[B20] Bosi S Feruglio L Milic D Prato M Synthesis and water solubility of novel fullerene bisadduct derivatives Eur J Org Chem 2003 24 4741 4747 10.1002/ejoc.200300494

[B21] Mosmann TJ Rapid colorimetric assay for cellular growth and survival: application to proliferation and cytotoxicity assays Inmunol Methods 1983 65 55 63 10.1016/0022-1759(83)90303-4 6606682

[B22] Higuchi R Fockler C Dollinger G Watson R Kinetic PCR analysis: real-time monitoring of DNA amplification reactions Biotechnology 1993 11 1026 1030 10.1038/nbt0993-1026 7764001

[B23] Giust D León D Ballesteros-Yañez I Da Ros T Albasanz JL Martín M Modulation of adenosine receptors by [60]fullerene hydrosoluble derivative in SK-N-MC cells ACS Chem Neurosci 2011 2 363 369 10.1021/cn200016q 22816023 PMC3369736

[B24] Giust D Da Ros T Martín M Albasanz JL Modulation of gene expression of adenosine and metabotropic glutamate receptors in rat’s neuronal cells exposed to L-glutamate and [60]fullerene J Biomed Nanotechnol 2014 10 1610 1619 10.1166/jbn.2014.1845 25016660

[B25] Albasanz JL Dalfó E Ferrer I Martín M Impaired metabotropic glutamate receptor/phospholipase C signaling pathway in the cerebral cortex in Alzheimer’s disease and dementia with Lewy bodies correlates with stage of Alzheimer’s-disease-related changes Neurobiol Dis 2005 20 685 693 10.1016/j.nbd.2005.05.001 15949941

[B26] Albasanz JL Rodriguez A Ferrer I Martín M Adenosine A_2A_ receptors are up-regulated in Pick’s disease frontal cortex Brain Pathol 2006 16 249 255 10.1111/j.1750-3639.2006.00026.x 17107593 PMC8095809

[B27] Albasanz JL Perez S Barrachina M Ferrer I Martín M Up-regulation of adenosine receptors in the frontal cortex in Alzheimer’s disease Brain Pathol 2008 18 211 219 10.1111/j.1750-3639.2007.00112.x 18241242 PMC8095610

[B28] Ribeiro JA Almeida AM Namorado JM Adenosine and adenosine triphosphate decrease ^45^Ca uptake by synaptosomes stimulated by potassium Biochem Pharmacol 1979 28 1297 1300 10.1016/0006-2952(79)90428-3 444297

[B29] de Mendonça A Sebastiao AM Ribeiro JA Inhibition of NMDA receptor-mediated currents in isolated rat hippocampal neurones by adenosine A1 receptor activation Neuroreport 1995 6 1097 1100 10.1097/00001756-199505300-00006 7662885

[B30] Arrigoni E Crocker AJ Saper CB Greene RW Scammell TE Deletion of presynaptic adenosine A_1_ receptors impairs the recovery of synaptic transmission after hypoxia Neuroscience 2005 132 575 580 10.1016/j.neuroscience.2004.12.009 15837119 PMC2259447

[B31] Castillo CA León D Ruíz MA Albasanz JL Martín M Modulation of adenosine A1 and A2A receptors in C6 glioma cells during hypoxia: involvement of endogenous adenosine J Neurochem 2008 105 2315 2329 10.1111/j.1471-4159.2008.05314.x 18315561

[B32] Cieślak M Komoszyński M Wojtcza A Adenosine A(2A) receptors in Parkinson’s disease treatment Purinergic Signalling 2008 4 305 312 10.1007/s11302-008-9100-8 18438720 PMC2583202

[B33] Matos M1 Augusto E Santos-Rodrigues AD Schwarzschild MA Chen JF Cunha RA Agostinho P Adenosine A2A receptors modulate glutamate uptake in cultured astrocytes and gliosomes Glia 2012 60 702 716 10.1002/glia.22290 22298379

[B34] Pshenichkin S Dolinska M Klauzinska M Luchenko V Grajkowska E Wroblewski JT Dual neurotoxic and neuroprotective role of metabotropic glutamate receptor 1 in conditions of trophic deprivation - possible role as a dependence receptor Neuropharmacology 2008 55 500 508 10.1016/j.neuropharm.2008.06.039 18619982 PMC2606294

[B35] Endoh T Characterization of modulatory effects of postsynaptic metabotropic glutamate receptors on calcium currents in rat nucleus tractus solitarius Brain Res 2004 1024 212 224 10.1016/j.brainres.2004.07.074 15451384

[B36] Moroni F Attucci S Cozzi A Meli E Picca R Scheideler MA Pellicciari R Noe C Sarichelou I Pellegrini-Giampietro DE The novel and systemically active metabotropic glutamate 1 (mGlu1) receptor antagonist 3-MATIDA reduces post-ischemic neuronal death Neuropharmacology 2002 42 741 751 10.1016/S0028-3908(02)00033-3 12015200

[B37] Lea PM Custera SJ Vicinia S Fade AI Neuronal and glial mGluR5 modulation prevents stretch-induced enhancement of NMDA receptor current Pharmacol Biochem Behav 2002 73 287 298 10.1016/S0091-3057(02)00825-0 12117582

